# Real-World Early Outcomes of the Avalus Aortic Valve: Results From 1250 Patients in the Avalus Clinical confidencE Registry

**DOI:** 10.1016/j.atssr.2026.02.037

**Published:** 2026-03-20

**Authors:** Lennart Vanglabeke, Tom Verbelen, Jean-Christian Roussel, Christian Lildal Carranza, Lenard Conradi, Koen Cathenis, Giovanni Troise, Julien Guihaire, Juan Bustamante-Munguira, Bart Meuris, ACE investigators, Johannes Petersen, Jules François, Jelle Fleerakkers, Maïra Gaillard, Aurélien Vallée, Adolfo Arévalo Abascal, Eduardo Velasco García, Davide Pacini, Carlo Mariani, Gianluca Folesani, Paolo Centofanti, Vittoria Lodo, Enrico Giuseppe Italiano, Nicolas Doll, Jochen Pöling, Mahmoud Wehbe, Rafael Llorens, Javier Estigarribia, Yanai Ben-Gal, Ariel Farkash, Amit Gordon, Francesco Musumeci, Federico Ranocchi, Antonio Lio, Tine Philipsen, Laurent de Kerchove, Gebrine El Khoury, Roberto Lorusso, Bart Maesen, Luca Aarts, Alberto Giovanni Tripodi, Alberto Canziani, Lorenzo Menicanti, Gianpiero Buttiglione, Antti Valtola, Jean Defraigne, Ruggero De Paulis, Louis Labrousse, Chris Van Kerrebroeck, Andreas Liebold, Leonid Sternik, Gonçalo Freitas Coutinho

**Affiliations:** 4 Department of Cardiovascular Surgery, Universitäres Herz- und Gefäßzentrum Hamburg, Hamburg, Germany; 5 Department of Cardiac Surgery, AZ Maria Middelares Ghent, Ghent, Belgium; 7 Department of Cardiac Surgery and Transplantation, Hôpital Marie Lannelongue, Groupe Hospitalier Paris Saint-Joseph, Université Paris Saclay, France; 8 Department of Cardiovascular Surgery, Hospital clinico de Valladolid, Valladolid, Spain; 9 Department of Cardiac Surgery, Osp. S.Orsola Malpighi - Bologna, Bologna, Italy; 10 Department of Cardiac Surgery, A. O. Ordine Mauriziano - Torino, Torino, Italy; 11 Department of Cardiac Surgery, Schüchtermann-Klinik, Bad Rothenfelde, Germany; 12 Department of Cardiovascular Surgery, Hospital Rambla, Tenerife, Spain; 13 Department of Cardiothoracic Surgery, Tel Aviv Medical Center, Tel Aviv, Israel; 14 Department of Cardiovascular Surgery, San Camillo Hospital Roma, Roma, Italy; 15 Department of Cardiac Surgery, UZ Gent, Gent, Belgium; 16 Department of Cardiothoracic and Vascular Surgery, UCL St-Luc - Bruxelles, Bruxelles, Belgium; 17 Department of Cardiothoracic Surgery, UMC Maastricht, Maastricht, the Netherlands & Cardiovascular Research Institute Maastricht (CARIM), Maastricht, the Netherlands; 18 Department of Cardiovascular Surgery, Villa Maria Cecilia-Cotignola, Cotignola, Italy; 19 Department of Cardiac Surgery, Policlinico San Donato S.P.A. - San Donato Milanese, Milan, Italy; 20 Department of Cardiac Surgery, Kuopio University Hospital, Kuopio, Finland; 21 Department of Cardiovascular and Thoracic Surgery, University Hospital of Liège, Liège, Belgium; 22 Cardiac Surgery Unit, European Hospital of Rome, Roma, Italy; 23 Department of Cardiology and Cardio-Vascular Surgery, Hopital Cardiologique de Haut-Leveque, Bordeaux University Hospital, Bordeaux, France; 24 Department of Cardiovascular Surgery, Ziekenhuis Oost-Limburg, Genk, Belgium; 25 Department of Cardiothoracic and Vascular Surgery, Ulm University, Ulm, Germany; 26 Department of Cardiac Surgery, The Leviev Cardiothoracic and Vascular Center, Sheba Medical Center, Tel Hashomer, Israel; 27 Department of Cardiothoracic Surgery, University of Coimbra, Coimbra, Portugal; 1 Department of Cardiac Surgery, University Hospitals Leuven, Belgium; 2 Department of Cardiovascular Sciences, KU Leuven, Leuven Belgium; 3 Department of Thoracic and Cardiovascular Surgery, CHU Nantes, Nantes, France; 4 Department of Cardiothoracic Surgery, Rigshospitalet København Ø, Copenhagen, Denmark; 5 Department of Cardiac Surgery, Universitätsklinikum Köln, Cologne, Germany; 6 Department of Cardiac Surgery, AZ Maria Middelares Ghent, Ghent, Belgium; 7 Department of Cardiac Surgery, Fondazione Poliambulanza Brescia, Brescia, Italy; 8 Department of Cardiac Surgery and Transplantation, Hôpital Marie Lannelongue, Université Paris Saclay, Paris, France; 9 Department of Cardiovascular Surgery, Hospital clinico de Valladolid, Valladolid, Spain

## Abstract

**Background:**

As cardiac surgery becomes more complex and patient profiles heterogeneous, real-world data are essential to evaluate bioprosthetic valve performance beyond controlled trials. The Avalus Clinical confidencE (ACE) registry evaluates safety and hemodynamic performance of the Avalus (Medtronic) bioprosthesis.

**Methods:**

The ACE registry is a prospective, multicenter, single-arm, observational study including 1250 adult patients undergoing surgical aortic valve replacement with the Avalus valve in 27 European centers (2021-2024). Exclusion criteria were age <18 years and salvage surgery. Baseline characteristics, procedural details, and outcomes were collected. Echocardiography was performed at discharge and at 1 year.

**Results:**

Mean age was 71.5 ± 6.5years, 22.2% female, and mean European System for Cardiac Operative Risk Evaluation-II was 3.5 ± 5.7. Isolated surgical aortic valve replacement was performed in 39.8% of patients; the remainder underwent combined procedures, most common was coronary artery bypass grafting (26%). Prosthesis sizes ranged from 19-29 mm, with a mean of 24.0 ± 2.2 mm; 18% of valves were 19-21 mm. Mean effective orifice area increased from 0.96 ± 0.63 cm^2^ preoperatively to 1.99 ± 0.60 cm^2^ at discharge and 1.80 ± 0.37 cm^2^ at 1 year. Mean gradients were low (discharge, 11.4 ± 5.1 mm Hg; 1 year, 12.2 ± 5.0 mm Hg). Patient-prosthesis mismatch rates at discharge were 20.8% moderate, and 4.8% severe; at 1 year, mismatch rates were 20.6% moderate, and 2.6% severe.

**Conclusions:**

In this 1250-patient, real-world registry, the Avalus valve demonstrated low gradients, favorable effective orifice area, and low severe patient-prosthesis mismatch rates, with reassuring early clinical outcomes across the spectrum of prosthesis sizes.

## Visual Abstract

**Figure:**
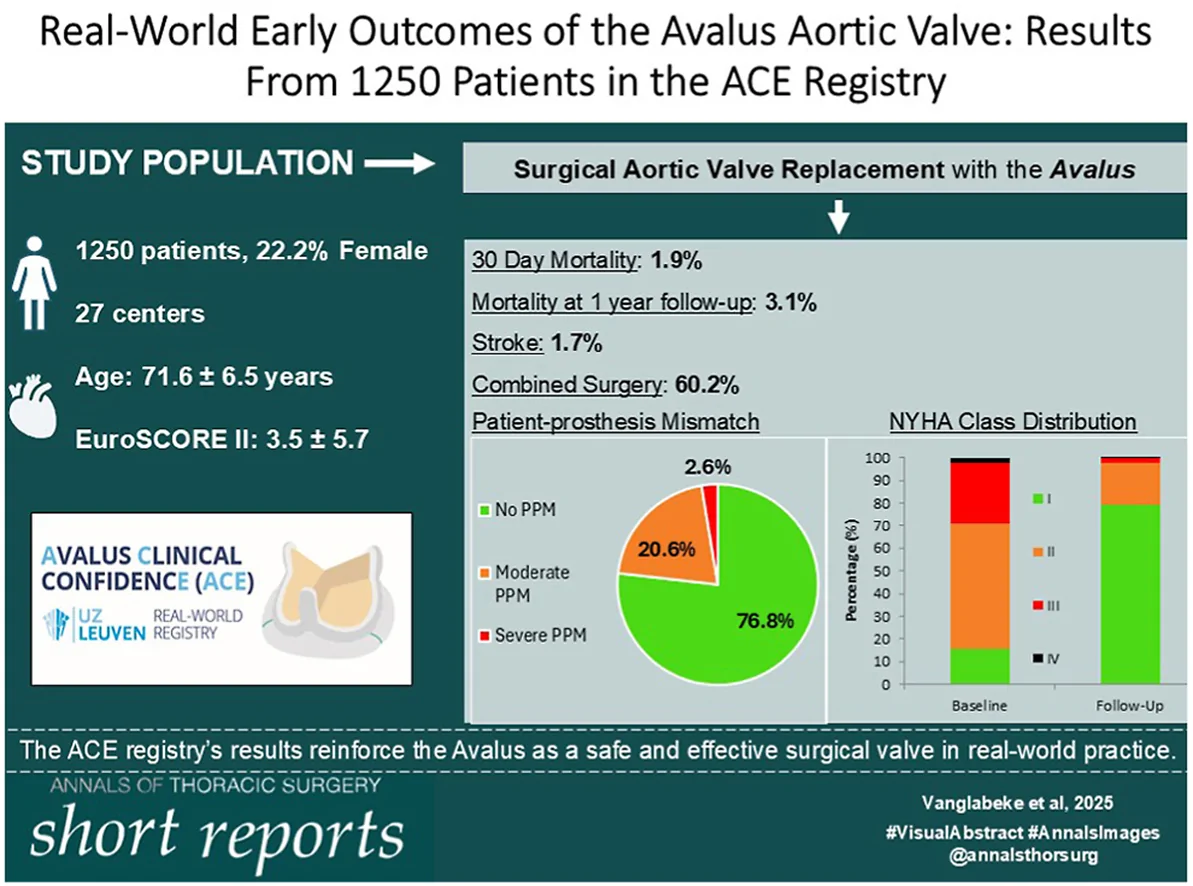

**In Short**

▪ Early outcomes demonstrated consistently low transvalvular gradients and favorable hemodynamic performance across the full Avalus (Medtronic) size range.
▪ Severe patient-prosthesis mismatch remained uncommon, even among patients receiving small valve sizes.

The surgical treatment of aortic valve disease is evolving within an increasingly complex environment. Patients undergoing aortic valve replacement present with advanced age, multiple comorbidities, and need for concomitant procedures.^1^ Concurrently, transcatheter aortic valve replacement has expanded treatment options, leading to renewed scrutiny of surgical bioprosthesis performance in truly representative populations.^2^ Real-world data, as opposed to controlled trials, are therefore essential to understand how contemporary valves function in routine care.^3^

The Avalus valve (Medtronic) is a stented bovine pericardial bioprosthesis designed with relatively large true internal diameters, aiming to optimize effective orifice area (EOA) and reduce the risk of patient-prosthesis mismatch (PPM).^4^ Evidence from the PERIcardial SurGical AOrtic Valve ReplacemeNt (PERIGON) pivotal-trial has demonstrated favorable hemodynamics and acceptable durability in selected patients undergoing isolated surgical aortic valve replacement (SAVR).^5^ However, broader real-world experience, including combined procedures and more complex risk profiles, has been less extensively described.

The Avalus Clinical confidencE (ACE) registry was established to evaluate the performance of the Avalus valve in 1250 patients in routine European practice. This report describes baseline and procedural characteristics in an “all-comers” population, early clinical outcomes, and valve hemodynamic performance.

## Patients and Methods

### Study Design and Population

The ACE registry is a prospective, multicenter, single-arm, observational study enrolling adults undergoing SAVR with the Avalus valve in 27 European centers. Between 2021 and 2024, 1250 patients were included. Exclusion criteria were limited to age <18 years and salvage procedures, allowing for an “all-comers” cohort with both isolated and combined procedures.^6^

All patients provided written informed consent for participation. The study was approved by the ethics committee of University Hospitals Leuven (lead center) and by local committees at participating institutions. The registry is registered (NCT05572710).

### Valve and Surgical Procedures

The Avalus valve is a stented bovine pericardial bioprosthesis with a supraannular design and alpha-amino-oleic-acid as anticalcification treatment. Valves are available in sizes 19-29 mm.^4^ Implantation followed local practice, with surgeon discretion regarding concomitant procedures and cardiopulmonary bypass strategy.

### Data Collection and Endpoints

Baseline clinical, echocardiographic, and surgical data were prospectively collected via electronic case report forms and stored in a secure REDCap database. Preoperative echocardiography included aortic valve area, transvalvular gradients, and left ventricular ejection fraction; follow-up was performed at discharge and 1 year.

The primary endpoint was the composite of all-cause death and disabling stroke; crude incidences of death, any stroke, new permanent pacemaker implantation, and valve-related rehospitalization are reported through available follow-up.

### Echocardiographic Assessment and PPM

Transthoracic echocardiography was performed according to local standards at discharge and at 1-year follow-up. Analyses included mean and peak transvalvular gradients and (indexed) EOA (iEOA). Because EOA was missing in a relevant subset of patients, multiple imputation was performed to generate a complete EOA and iEOA dataset.

PPM was classified according to body mass index (BMI)-adjusted Valve Academic Research Consortium 3 criteria.^6^ In nonobese patients (BMI <30 kg/m^2^), severe and moderate PPM were defined as iEOA≤0.55 cm^2^/m^2^ and 0.56-0.85 cm^2^/m^2^, respectively; in obese patients (BMI ≥30 kg/m^2^), corresponding thresholds were ≤0.70 cm^2^/m^2^ and 0.71-0.90 cm^2^/m^2^.

### Statistical Analysis

Continuous variables are reported as mean ± SD and categorical variables as counts and percentages; missing EOA values were addressed using multiple imputation.

## Results

### Baseline Characteristics

Baseline characteristics are summarized in Table 1. Mean age was 71.5 ± 6.5 (range, 42-92) years; 11.9% of patients were aged <65 years. Women accounted for 22.2% of the cohort. Mean BMI was 27.6 ± 4.6 kg/m^2^ and mean body surface area was 1.95 ± 0.21 m^2^. More than half of patients had at least moderate renal impairment: 43.4% had creatinine clearance 50-85 mL/min and 9.3% <50 mL/min.

**Table 1:** Baseline and Procedural Characteristics of 1250 Patients Undergoing AVR

Characteristic	Patients (N = 1250)
Age, y	71.6 ± 6.5
Range	42-92
<65y	149 (11.9)
Female sex	278/1250 (22.2)
BMI, kg/m^2^	27.6 ± 4.7
BSA, m^2^	1.95 ± 0.2
Renal impairment	
Moderate	542/1242 (43.4)
Severe	116/1242 (9.3)
NYHA class	
I-II	877/1233 (71.1)
III-IV	356/1233 (28.9)
EuroSCORE-II, %	3.5 ± 5.7
Range	0.52-77.4
Echocardiography	
Left ventricular ejection fraction, %	56.7 ± 10.1
Aortic valve area, cm^2^	0.96 ± 0.6
Mean aortic valve gradient, mm Hg	41.3 ± 20
Peak aortic valve gradient, mm Hg	62.7 ± 30.1
Aortic regurgitation	
Mild	404/1237 (32.7)
Moderate	219/1237 (17.7)
Severe	273/1237 (22.1)
Urgency	
Urgent	155/1250 (12.4)
Emergent	12/1200 (1.0)
Bicuspid valve	324/1250 (25.9)
Single AVR	497/1248 (39.8)
Concomitant procedures	
CABG	325/1250 (26.0)
Mitral valve	112/1250 (9.0)
Tricuspid valve	48/1250 (3.8)
Ascending aorta	208/1250 (16.6)
Ablation	80/1250 (6.4)
Aortic cross-clamp time, min	89.2 ± 32.1
CPB time, min	119.1 ± 45.2

Values are presented as mean ± SD, n (%), or n/N (%), unless otherwise noted.

AVR, aortic valve replacement; BMI, body mass index; BSA, body surface area; CABG, coronary artery bypass grafting; CPB, cardiopulmonary bypass; EuroSCORE, European System for Cardiac Operative Risk Evaluation; NYHA, New York Health Association.

Heart failure symptoms were common: 28.9% of patients were in New York Heart Association (NYHA) class III–IV preoperatively. The mean European System for Cardiac Operative Risk Evaluation (EuroSCORE)-II was 3.5 ± 5.7, reflecting a broad risk spectrum including some higher-risk patients. Preoperative left ventricle ejection fraction averaged 56.7% ± 10.1%.

The predominant lesion was aortic stenosis, with a mean preoperative aortic valve area of 0.96 ± 0.6 cm^2^ and mean gradient 41.3 ± 20 mm Hg; peak gradient was 62.7 ± 30.1 mm Hg. Aortic regurgitation of varying degrees was present, and mixed valve disease was frequent, consistent with real-world practice.

### Procedural Characteristics

**Figure 1:**
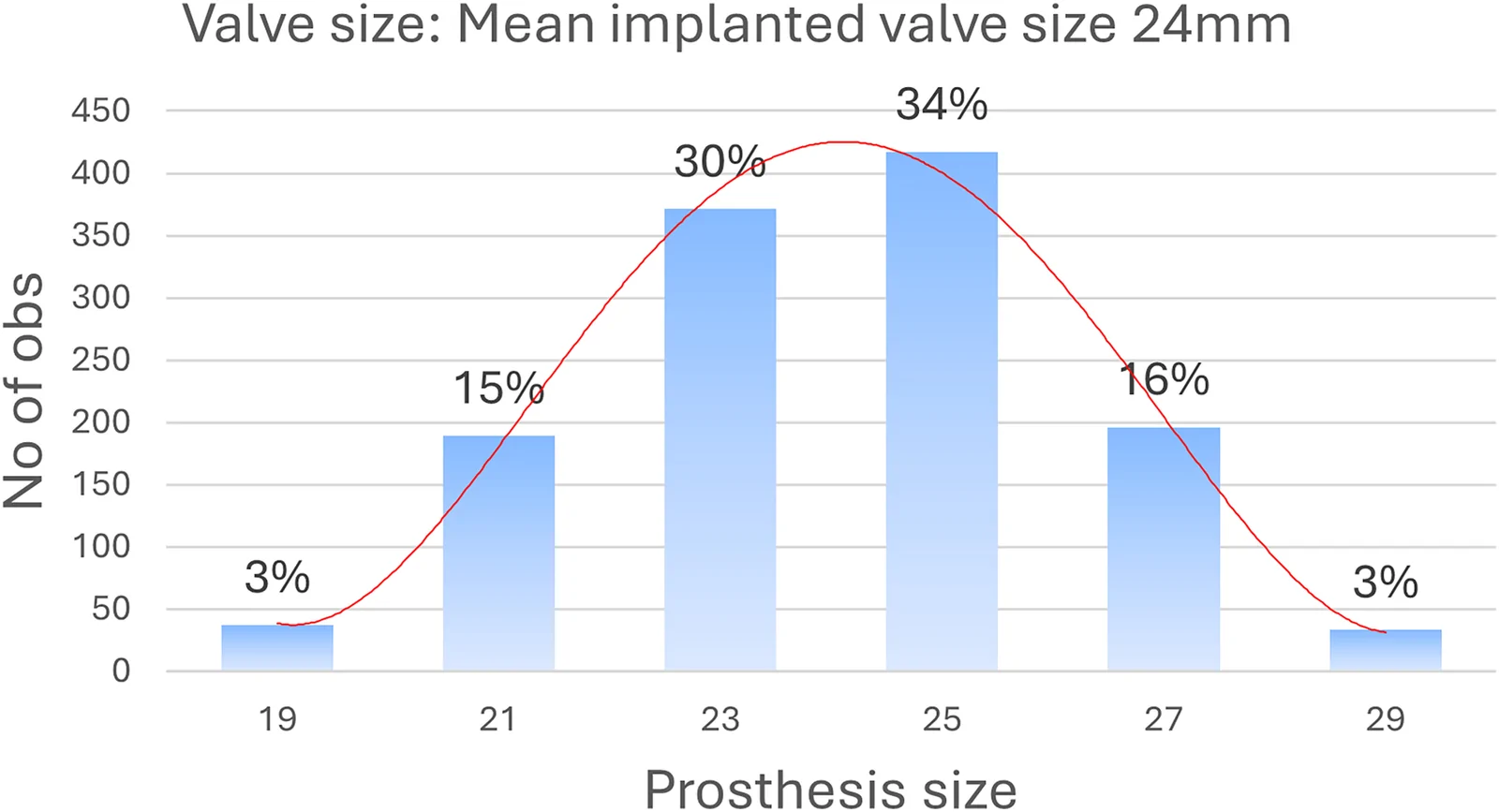
Distribution of prosthesis sizes in the Avalus Clinical confidencE registry cohort. (obs, observations.)

Procedural details are presented in Table 1. Prosthesis sizes ranged from 19-29 mm (Figure 1). The mean labeled prosthesis size was 24.0 mm; 18% were 19 or 21 mm, reflecting small annuli inclusion.

Isolated SAVR was performed in 39.8% of patients, with the remainder undergoing concomitant procedures. Coronary artery bypass grafting was performed in approximately 26% of the cohort, mitral and/or tricuspid valve surgery in 9% and 3.8%, respectively, ascending aortic replacement in 16.6%, and ablation procedures in 6.4%. Reflecting this procedural complexity, mean cardiopulmonary bypass time was 119.1 minutes.

Extra baseline and procedural characteristics can be found in the Supplemental Material.

### Clinical Outcomes

Through available follow-up (1 year), any recorded death occurred in 45 of 1250 patients (3.6%). Stroke (transient ischemic attack and cerebrovascular accident) was documented in 39 patients (3.1%). New permanent pacemaker implantation during the index hospitalization or follow-up was required in 5.7% of patients, and rehospitalization for valve-related symptoms in 3.7%.

Intensive care unit stay averaged 3.1 ± 5.9 days, with prolonged ventilation, need for dialysis, and mechanical circulatory support required only in a minority of cases. Among patients with available 1-year functional status, 97.5% were in NYHA-class I-II, indicating substantial symptomatic improvement from baseline.

Clinical and echocardiographic outcomes are summarized in Table 2.

**Table 2:** Clinical Outcomes and Echocardiographic Performance at Discharge and 1-Year Follow-up

Characteristic	30 d (discharge) (N = 1250)	1 y (follow-up) (n = 673)
Mortality	24/1250 (1.9)	21/673 (3.1)
Cardiac cause	9/24 (37.5)	5/21 (23.8)
Thromboembolic event	28/1229 (2.3)	11/648 (1.7)
TIA	10/28 (35.7)	3/11 (27.3)
Ischemic	6/10 (60.0)	2/3 (66.7)
Undetermined	4/10 (40.0)	1/3 (33.3)
CVA	18/28 (64.3)	8/11 (72.7)
Ischemic	11/18 (61.1)	4/8 (50.0)
Hemorrhagic	2/18 (11.1)	1/8 (12.5)
Undetermined	3/18 (16.7)	3/8 (37.5)
NYHA class		
I-II		605/620 (97.6)
III-IV		15/620 (2.4)
Postoperative pacemaker implantation	52/1250 (4.1)	19/645 (2.9)
Acute kidney injury		
Stage 2	32/1221 (2.6)	
Stage 3	28/1221 (2.3)	
Need for dialysis	26/1233 (2.1)	
ICU duration, d	3.1 ± 5.9	
Hospital duration, d	10.8 ± 9.4	
Valve thrombosis		0/645 (0.0)
Structural valve deterioration		0/619 (0.0)
Bleeding	111/1144 (8.8)	7/647 (1.1)
Minor	40/111 (36.0)	0/7 (0.0)
Major	51/111 (45.9)	4/7 (57.1)
Life-threatening	20/111 (18.0)	3/7 (42.9)
Rehospitalization for valve-related symptoms		24/645 (3.7)
Cardiac reoperation	71/1235 (5.7)	17/651 (2.6)
Valve		7/17 (41.2)
Endocarditis		8/17 (47.1)
Bleeding	49/71 (69.0)	0/17 (0.0)
Echocardiographic findings		
Left ventricular ejection fraction, %	54.7 ± 10.0	58.2 ± 8.6
Aortic valve area, cm^2^	1.99 ± 0.6	1.80 ± 0.5
Mean aortic valve gradient, mm Hg	11.6 ± 5.2	12.2 ± 5.0
Peak aortic valve gradient, mm Hg	20.7 ± 8.7	20.6 ± 8.6
Aortic regurgitation		
Intravalvular		
Mild	41/1250 (3.3)	24/577 (4.2)
Severe	1/1250 (0.1)	
Paravalvular		
Mild	28/1250 (2.2)	13/577 (2.3)
Moderate	3/1250 (0.2)	
PPM		
Mild	260/1250 (20.8)	257/1250 (20.6)
Severe	60/1250 (4.8)	33/1250 (2.6)

Values are presented as mean ± SD, n (%), or n/N (%), unless otherwise noted.

CVA, cerebrovascular accident; ICU, intensive care unit; NYHA, New York Health Association; PPM, patient-prosthesis mismatch; TIA, transient ischemic attack.

At discharge, mean EOA increased to 1.99 ± 0.60 cm^2^ from a preoperative mean of 0.96 ± 0.63 cm^2^, with a mean iEOA of 1.03 ± 0.32 cm^2^/m^2^. Mean and peak transvalvular gradients at discharge were 11.6 ± 5.2 mmHg and 20.7 ± 8.7 mm Hg, respectively, reflecting favorable early hemodynamic performance, including in smaller valve sizes.

**Figure 2:**
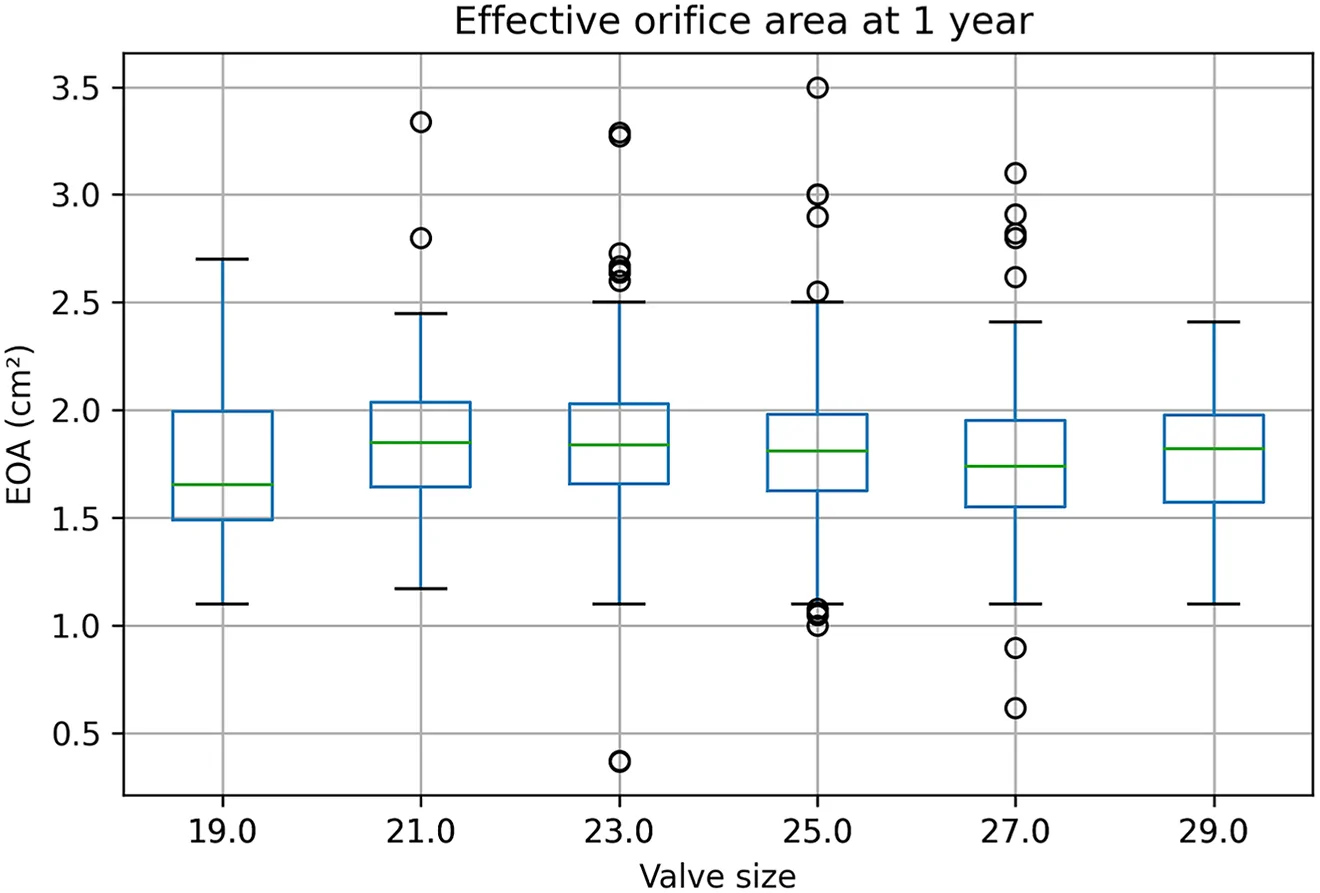
Hemodynamic performance at 1 year according to valve size. (EOA, effective orifice area.)

At 1 year, mean EOA was 1.80 ± 0.5 cm^2^ and mean iEOA 0.93 ± 0.19 cm^2^/m^2^. Mean gradients remained low at 12.2 ± 5.0 mm Hg with peak gradients at 20.6 ± 8.6 mm Hg, suggesting stable hemodynamics over the first year (Figure 2).

PPM prevalence was favorable. At discharge, 74.4% of patients had no PPM, 20.8% moderate, and 4.8% severe. At 1 year, the corresponding proportions were 76.8%, 20.6%, and 2.6%, respectively. Severe PPM remained uncommon at both time points, with the majority of patients classified as having no PPM.

More clinical outcomes can be found in the Supplemental Material.

## Comment

In this registry, the Avalus bioprosthesis demonstrated low early mortality, acceptable rates of stroke and other valve-related adverse events, and excellent hemodynamic performance across the full range of valve sizes.

These findings reinforce earlier trial and registry data for Avalus. The PERIGON pivotal trial and subsequent long-term analyses have shown sustained hemodynamic performance and encouraging survival in more selected populations. The ACE registry, by contrast, includes a broad “all-comers” cohort with combined procedures, advanced age, and a wide risk spectrum. Within this complex environment, the valve maintained low gradients and favorable iEOA, suggesting that design features such as a relatively large true internal diameter translate into real-world benefit.^7^

PPM remains a concern after SAVR, particularly in small annuli, where traditional valves can yield high gradients and undersized EOAs. Reports have cited moderate PPM rates exceeding 50% and severe PPM in up to one third of patients with certain prostheses.^8^^,^^9^ In this registry, combined moderate/severe PPM affected 25.6% of patients at discharge and 23.2% at 1 year, while severe PPM was uncommon. Notably, this pattern was observed including a meaningful proportion of 19-21 mm implants, suggesting that acceptable early hemodynamics were generally achieved across the entire size range included in this cohort.

Clinically, mortality and stroke rates through approximately 1 year compare favorably with predicted risk and with historical SAVR series. The relatively low need for permanent pacemaker implantation and valve-related rehospitalization support the safety profile of the valve in contemporary practice. The consistent improvement in NYHA class aligns with the observed hemodynamic gains.

### Limitations

This analysis has limitations inherent to its observational design. First, the absence of a randomized comparator precludes direct comparisons with other bioprostheses. Second, echocardiography was performed according to local practice without core laboratory adjudication, and missing EOA data required imputation. Third, follow-up echocardiography was incomplete at the time of analysis; 1-year hemodynamic findings therefore reflect a subset of the full cohort. Finally, this report focuses on early outcomes; longer-term durability, structural valve deterioration, and late reintervention rates will require continued registry follow-up.

### Conclusions

In a 1250-patient, multicenter, real-world registry of SAVR with the Avalus valve, early mortality/stroke rates were low, hemodynamics were excellent, and severe PPM was uncommon. Mean gradients remained low and EOA was preserved through 1 year, with most patients experiencing symptomatic improvement. These findings support the Avalus valve as a reliable option in contemporary practice, with ongoing ACE-registry follow-up informing long-term outcomes.

## References

[1] Pierri M.D., Capestro F., Zingaro C., Torracca L. (2010). The changing face of cardiac surgery patients: an insight into a Mediterranean region. Eur J Cardiothorac Surg.

[2] Sigala E., Kelesi M., Terentes-Printzios D. (2023). Surgical aortic valve replacement in patients aged 50 to 70 years: mechanical or bioprosthetic valve? A systematic review. Healthc.

[3] Franklin J.M., Schneeweiss S. (2017). When and how can real world data analyses substitute for randomized controlled trials?. Clin Pharmacol Ther.

[4] Avalus bioprosthesis for heart valve replacement Medtronic. https://europe.medtronic.com/xd-en/healthcare-professionals/products/cardiovascular/heart-valves-surgical/avalus-bioprostheses.html.

[5] Sabik J.F., Rao V., Dagenais F. (2024). Seven-year outcomes after surgical aortic valve replacement with a stented bovine pericardial bioprosthesis in over 1100 patients: a prospective multicentre analysis. Eur J Cardiothorac Surg.

[6] Dismorr M., Glaser N., Franco-Cereceda A., Sartipy U. (2023). Effect of prosthesis-patient mismatch on long-term clinical outcomes after bioprosthetic aortic valve replacement. J Am Coll Cardiol.

[7] van Boxtel A.G.M., Mariani M.A., Ebels T. (2023). All surgical supra-annular aortic valvar tissue prostheses are labelled too large. Interdiscip CardioVasc Thorac Surg.

[8] Ternacle J., Théron A., Bernard A. (2024). Prosthesis-patient mismatch after aortic valve replacement: valve size matters?. J Heart Valve Soc.

[9] Clavel M.-A., Webb J.G., Pibarot P. (2009). Comparison of the hemodynamic performance of percutaneous and surgical bioprostheses for the treatment of severe aortic stenosis. J Am Coll Cardiol.

