# Supplementary material for: Real-World Early Outcomes of the Avalus Aortic Valve: Results From 1250 Patients in the Avalus Clinical confidencE Registry
Source: Ann Thorac Surg Short Rep. 2026 Mar 20;4(3):830–6. doi: 10.1016/j.atssr.2026.02.037 (PMC13555416; doi:10.1016/j.atssr.2026.02.037)
Supplement: Supplementary file 1 [file mmc1.docx]

# Supplemental tables

## S1: Baseline & Procedural characteristics

| Table S1. Baseline & Procedural characteristics | |
| --- | --- |
| Characteristic | **Patients (N=1250)** |
| Age (yr) | 71.6±6.5 |
| Age range | 42—92 |
| <65y (no.(%)) | 149 (11.9) |
| >75y (no.(%)) | 339 (27.1) |
| Female sex (no.(%)) | 278 (22.2) |
| BMI (kg/m²) | 27.6±4.7 |
| BSA (m²) | 1.95±0.2 |
| Renal impairment (no.(%)) |  |
| Normal | 584/1242 (47.3) |
| Moderate | 542/1242 (43.4) |
| Severe | 116/1242 (9.3) |
| NYHA class (no./total no.(%)) |  |
| I | 192/1233 (15.7) |
| II | 685/1233 (55.0) |
| III | 326/1233 (26.5) |
| IV | 30/1233 (2.8) |
| EuroSCORE II (%) | 3.5±5.7 |
| Median | 1.88 (IQR 1.14–3.71) |
| EuroSCORE II range | 0.52—77.4 |
| Clinical history (no./total no.(%)) |  |
| Diabetes: insulin treated | 76/1244 (6.1) |
| Previous cardiac surgery | 70/1238 (5.7) |
| Severe impairment of mobility | 26/1231 (2.1) |
| Chronic lung disease | 87/1230 (7.1) |
| Pulmonary hypertension |  |
| None | 952/1225 (77.7) |
| Moderate | 242/1225 (19.8) |
| Severe | 31/1225 (2.5) |
| Endocarditis | 57/1228 (4.6) |
| Critical preop state | 20/1241 (1.6) |
| Extra-cardiac arteriopathy | 103/1241 (8.3) |
| Recent myocardial infarction | 32/1237 (2.6) |
| Rhythm (no./total no.(%)) |  |
| Sinus rhythm | 1012/1239 (81.7) |
| Atrial fibrillation | 184/1239 (14.6) |
| Pacemaker | 36/1239 (2.9) |
| CCS class 4 angina | 32/1242 (2.6) |
| Echocardiographic findings |  |
| Left ventricular ejection fraction (%) | 56.7±10.1 |
| Aortic valve area (cm²) | 0.96±0.6 |
| Mean aortic valve gradient (mmHg) | 41.3±20.0 |
| Peak aortic valve gradient (mmHg) | 62.7±30.1 |
| Aortic regurgitation (no./total no.(%)) |  |
| Mild | 404/1237 (32.7) |
| Moderate | 219/1237 (17.7) |
| Severe | 273/1237 (22.1) |
| Prosthesis size (no./total no.(%)) |  |
| 19 | 37/1243 (3.0) |
| 21 | 188/1243 (15.1) |
| 23 | 372/1243 (29.6) |
| 25 | 417/1243 (33.5) |
| 27 | 196/1243 (15.8) |
| 29 | 33/1243 (2.7) |
| Urgency (no./total no.(%)) |  |
| Elective | 1083/1250 (86.6) |
| Urgent | 155/1250 (12.4) |
| Emergent | 12/1200 (1.0) |
| Native valve (no./total no.(%)) |  |
| Unicuspid | 1/1250 (0.1) |
| Bicuspid | 324/1250 (25.9) |
| Tricuspid | 886/1250 (70.9) |
| Prosthetic | 33/1250 (2.6) |
| Single AVR (no./total no.(%)) | 497/1248 (39.8) |
| Concomitant procedures (no./total no.(%)) |  |
| CABG | 325/1250 (26.0) |
| Number of grafts | 1.77±0.9 |
| Graft range | 0—5 |
| Mitral valve repair/replacement | 112/1250 (9.0) |
| Tricuspid valve repair/replacement | 48/1250 (3.8) |
| Ascending aorta aneurysm | 208/1250 (16.6) |
| Ablation treatment | 80/1250 (6.4) |
| LAAO | 7/1250 (0.6) |
| Other | 202/1250 (16.2) |
| Aortic cross-clamp time (minutes) | 89.2±32.1 |
| Isolated AVR | 66.0 (IQR 55.0–80.0) |
| Combined procedures | 97.0 (IQR 78.0–118.0) |
| Second clamp needed \| no./total no.(%)) |  |
| Paravalvular leak | 4/1249 (0.3) |
| Bleeding | 7/1249 (0.6) |
| CPB time (minutes) | 119.1±45.2 |
| Isolated AVR | 88.0 (IQR 73.0–105.0) |
| Combined procedures | 126.0 (IQR 104.0–159.0) |
| Access (no./total no.(%)) |  |
| Full sternotomy | 1038/1242 (83.6) |
| Mini sternotomy | 170/1242 (13.7) |
| Anterior thoracotomy | 22/1242 (1.8) |
| Use of automatic suturing device (no./total no.(%)) | 6/165 (3.6) |

*Table 1: Patient baseline characteristics & procedural characteristics. AVR, Aortic Valve Replacement; BMI, Body Mass Index; BSA, Body Surface Area; CABG, Coronary Artery Bypass Graft*; *CCS, Canadian classification Cardiovascular Society; CPB, Cardiopulmonary Bypass; EuroSCORE, European System for Cardiac Operative Risk Evaluation; IQR, Interquartile Range; NYHA, New York Heart Association.*

## S2: Status at discharge and 1-year follow-up

| Table 3. Status at discharge and 1-year follow-up | |  |
| --- | --- | --- |
| Characteristic | **Discharge (N=1250)** | **Follow-up (N=673)** |
| Mortality (no./total no.(%)) | 24/1250 (1.9) | 21/673 (3.1) |
| Cardiac cause | 9/24 (37.5) | 5/21 (23.8) |
| Procedure-related | 1/24 (4.2) | 0/21 (0.0) |
| Sudden or unwitnessed death | 2/24 (8.3) | 0/21 (0.0) |
| Other | 12/24 (50.0) | 16/21 (76.2) |
| Stroke (no./total no.(%)) | 28/1229 (2.3) | 11/648 (1.7) |
| TIA | 10/28 (35.7) | 3/11 (27.3) |
| Ischemic | 6/10 (60.0) | 2/3 (66.7) |
| Undetermined | 4/10 (40.0) | 1/3 (33.3) |
| CVA | 18/28 (64.3) | 8/11 (72.7) |
| Ischemic | 11/18 (61.1) | 4/8 (50.0) |
| Haemorrhagic | 2/18 (11.1) | 1/8 (12.5) |
| Undetermined | 3/18 (16.7) | 3/8 (37.5) |
| Periprocedural myocardial infarction (no./total no.(%)) | 6/1230 (0.5) |  |
| Cardiac reoperation (no./total no.(%)) | 71/1235 (5.7) | 17/651 (2.6) |
| Bleeding | 49/71 (69.0) | 0/17 (0.0) |
| Valve |  | 7/17 (41.2) |
| Endocarditis |  | 8/17 (47.1) |
| Other | 22/71 (31.0) | 2/17 (11.8) |
| Bleeding (no./total no.(%)) | 111/1144 (8.8) | 7/647 (1.1) |
| Minor bleeding | 40/111 (36.0) | 0/7 (0.0) |
| Major bleeding | 51/111 (45.9) | 4/7 (57.1) |
| Life-threatening | 20/111 (18.0) | 3/7 (42.9) |
| Postoperative pacemaker implantation (no./total no.(%)) | 52/1250 (4.1) | 19/645 (2.9) |
| Acute kidney injury (no./total no.(%)) |  |  |
| Stage 1 | 97/1221 (7.9) |  |
| Stage 2 | 32/1221 (2.6) |  |
| Stage 3 | 28/1221 (2.3) |  |
| Need for dialysis (no./total no.(%)) | 26/1233 (2.1) |  |
| Prolonged ventilation (no./total no.(%)) | 73/1238 (5.9) |  |
| Need for mechanical support (no./total no.(%)) |  |  |
| ECMO | 6/1222 (0.4) |  |
| IABP | 7/1222 (0.6) |  |
| ICU duration (days) | 3.1±5.9 |  |
| Hospital duration (days) | 10.8±9.4 |  |
| Valve thrombosis |  | 0/645 (0.0) |
| Structural valve degeneration |  | 0/619 (0.0) |
| Re-hospitalization for valve-related symptoms |  | 24/645 (3.7) |
| Cardiac reoperation | 71/1235 (5.7) | 17/651 (2.6) |
| Valve |  | 7/17 (41.2) |
| Endocarditis |  | 8/17 (47.1) |
| Bleeding | 49/71 (69.0) | 0/17 (0.0) |
| Echocardiographic findings |  |  |
| Left ventricular ejection fraction (%) | 54.7±10.0 | 58.2±8.6 |
| Aortic valve area (cm²) | 1.99±0.6 | 1.80±0.5 |
| Mean aortic valve gradient (mmHg) | 11.6±5.2 | 12.2±5.0 |
| Peak aortic valve gradient (mmHg) | 20.7±8.7 | 20.6±8.6 |
| Aortic regurgitation (no./total no.(%)) |  |  |
| Intravalvular |  |  |
| Mild | 41/1250 (3.3) | 24/577 (4.2) |
| Severe | 1/1250 (0.1) |  |
| Paravalvular |  |  |
| Mild | 28/1250 (2.2) | 13/577 (2.3) |
| Moderate | 3/1250 (0.2) |  |
| PPM |  |  |
| No PPM | 930/1250 (74.4) | 960/1250 (76.8) |
| Mild PPM | 260/1250 (20.8)) | 257/1250 (20.6) |
| Severe PPM | 60/1250 (4.8) | 33/1250 (2.6) |
| Oral anticoagulants | 445/1221 (36.4) | 102/640 (15.9) |
| Novel oral anticoagulants | 229/1204 (19.0) | 176/641 (27.5) |
| Antiplatelet medication | 795/1208 (65.8) | 372/638 (58.3) |
| Rhythm (no./total no.(%)) |  |  |
| Sinus rhythm | 924/1221 (75.7) | 515/629 (81.9) |
| Atrial fibrillation | 215/1221 (17.6) | 74/629 (11.8) |
| Pacemaker | 64/1221 (5.2) | 25/629 (3.9) |
| NYHA class (no./total no.(%)) |  |  |
| I | 513/650 (78.9) | 492/620 (79.4) |
| II | 120/650 (18.5) | 113/620 (18.2) |
| III | 13/650 (2.0) | 13/620 (2.1) |
| IV | 2/650 (0.03) | 2/620 (0.3) |
| Rehospitalization for valve related symptoms (no./total no.(%)) |  | 24/645 (3.7) |

*Table S2: Status at discharge and 1-year follow-up. CVA, Cerebrovascular Accident; ECMO, ExtraCorporeal Membrane Oxygenation; IABP, Intra-Aortic Balloon Pump; NYHA, New York Heart Association; PPM, Patient-Prosthesis-Mismatch; TIA, Transient Ischemic Attack.*
